# One assay to test them all: Multiplex assays for expansion of respiratory virus surveillance

**DOI:** 10.3389/fmed.2023.1161268

**Published:** 2023-04-24

**Authors:** Narjis Boukli, Claude Flamand, Kim Lay Chea, Leangyi Heng, Seangmai Keo, Kimhoung Sour, Sophea In, Panha Chhim, Bunthea Chhor, Lomor Kruy, Jelena D. M. Feenstra, Manoj Gandhi, Obiageli Okafor, Camilla Ulekleiv, Heidi Auerswald, Viseth Srey Horm, Erik A. Karlsson

**Affiliations:** ^1^Virology Unit, National Influenza Center, WHO H5 Regional Reference Laboratory, World Health Organization COVID-19 Global Referral Laboratory, Institut Pasteur du Cambodge, Phnom Penh, Cambodia; ^2^Epidemiology and Public Health Unit, Institut Pasteur du Cambodge, Phnom Penh, Cambodia; ^3^Mathematical Modelling of Infectious Diseases Unit, Institut Pasteur, CNRS, Paris, France; ^4^Thermo Fisher Scientific, South San Francisco CA, United States

**Keywords:** SARS-CoV-2, influenza, respiratory syncytial virus, COVID-19, multiplex assay, diagnostic test, surveillance

## Abstract

Molecular multiplex assays (MPAs) for simultaneous detection of severe acute respiratory syndrome coronavirus 2 (SARS-CoV-2), influenza and respiratory syncytial virus (RSV) in a single RT-PCR reaction reduce time and increase efficiency to identify multiple pathogens with overlapping clinical presentation but different treatments or public health implications. Clinical performance of XpertXpress^®^ SARS-CoV-2/Flu/RSV (Cepheid, GX), TaqPath™ COVID−19, FluA/B, RSV Combo kit (Thermo Fisher Scientific, TP), and PowerChek™ SARS-CoV-2/Influenza A&B/RSV Multiplex RT-PCR kit II (KogeneBiotech, PC) was compared to individual Standards of Care (SoC). Thirteen isolates of SARS-CoV-2, human seasonal influenza, and avian influenza served to assess limit of detection (LoD). Then, positive and negative residual nasopharyngeal specimens, collected under public health surveillance and pandemic response served for evaluation. Subsequently, comparison of effectiveness was assessed. The three MPAs confidently detect all lineages of SARS-CoV-2 and influenza viruses. MPA-LoDs vary from 1 to 2 Log10 differences from SoC depending on assay and strain. Clinical evaluation resulted in overall agreement between 97 and 100%, demonstrating a high accuracy to detect all targets. Existing differences in costs, testing burden and implementation constraints influence the choice in primary or community settings. TP, PC and GX, reliably detect SARS-CoV-2, influenza and RSV simultaneously, with reduced time-to-results and simplified workflows. MPAs have the potential to enhance diagnostics, surveillance system, and epidemic response to drive policy on prevention and control of viral respiratory infections.

## 1. Introduction

Aside from novel coronavirus disease 2019 (COVID−19), respiratory infections with viral pathogens remain a major global burden (1–3). Since numerous respiratory viruses circulate concurrently with similar clinical presentations, diagnosis requires laboratory testing for several pathogens. Any delays in accurate and timely identification can compromise patient care (4).

Real-time polymerase chain reaction (RT-PCR) on upper respiratory tract (URT) swabs is the gold standard for diagnosis of viral respiratory infections (VRIs) (5). Between 2020 and 2022, public health measures to constrain COVID−19 significantly altered incidence of VRIs (6). However, with reduction of restrictions and fatigue over prevention behaviors, both influenza and respiratory syncytial virus (RSV) are resurging (7, 8). In addition, spillovers of novel zoonotic influenza viruses continually represent a human threat (9, 10). Cambodia is located in the tropics, an area traditionally considered to have a poorly defined influenza seasonality compared to temperate regions. However, while influenza is detected year round, Cambodia does have a specific influenza season generally peaking during the rainy season from June to November (11). Unfortunately, much less is known about RSV and SARS-CoV-2 circulation, especially in recent years. RSV is commonly detected in acute lower respiratory tract infections of young Cambodian children (12). The full seasonality of RSV and SARS-CoV-2 need to be further studied and employing a multiple pathogen approach will allow for better surveillance and understanding of seasonality and co-circulation. In addition, as co-infections are prevalent, especially in certain patients, e.g., children under 5 years (13), the simultaneous diagnostic of multiple respiratory viruses is advantageous due to saving time, cost and required sample volume. A accelerated detection of co-infections is also necessary as they can increase severity and mortality (14, 15) and demand rapid, specific treatment. Funding issues, disruptions in reagent procurement and supply chains, and inadequate human resources reduce diagnostic testing capacity, especially under pandemic conditions. Therefore, improvement of VRI surveillance needs to account not only for multiple pathogens and their potential genetic and seasonal changes, but also for human resources, capacity, and cost.

Molecular multiplex assays (MPAs) allowing detection of several pathogens in a single RT-PCR have demonstrated utility for diagnostics of influenza and RSV (16). Early in the COVID-19 pandemic, manufacturers modified existing MPAs to simultaneously detect severe acute respiratory syndrome coronavirus-2 (SARS-CoV-2) (5, 17). Integration of MPAs into public health surveillance strategies to detect SARS-CoV-2 and influenza has been widely discussed and implemented in countries such as in the United States and other jurisdictions such as Wales (18, 19). Considering the co-circulation of respiratory viruses and suggested expansion of testing in global surveillance, MPAs may be an attractive option (20, 21). However, viral evolution, genetic bottlenecks, and emergence of novel avian influenza (AIV) strains could impair viral detection (22, 23).

Comparison between MPAs and standard protocols allows evaluation of the clinical performance, as well as cost and testing burden for three commercial multiplex RT-PCR assays intended to simultaneously detect SARS-CoV-2, influenza, and RSV.

## 2. Materials and methods

### 2.1. Assays

Three MPAs available and easily implemented in Cambodia were performed according to manufacturers’ protocols (Table 1).

1. XpertXpress™ SARS-CoV-2/Flu/RSV test (GX) (Cepheid, CA, United States), a closed unitary MPA, integrates specimen extraction, RT-PCR, and target detection (24). A GeneXpert Xpress XVI-16 instrument (Cepheid) served to run cartridges, and instrument software generated result interpretation.
2. TaqPath™ COVID-19, FluA/B, RSV Combo Kit (TP) (Thermo Fisher Scientific, MA, United States) is a MPA with two targets for each virus (25). RT-PCR was performed on the QuantStudio™ 5 RT-PCR Instrument, 0.2 ml block (Applied Biosystems, MA, United States) and results were analyzed using the Pathogen Interpretive Software CE-IVD Edition v1.1.0 (Applied Biosystems).
3. PowerChek™ SARS-CoV-2, Influenza A&B, RSV Multiplex Real-time PCR Kit II (PC) (KogeneBiotech, Inchon, Korea), a MPA with one targeted gene for each virus (26), was performed on the CFX96™ RT-PCR Detection System (Bio-Rad Laboratories, CA, United States) and results analyzed with CFX96™ software.

**Table 1:** Genes targeted for each virus and each assay.

	SARS-CoV-2	Influenza A	Influenza B	RSV
SOC	E; RdRp^a^	M	M	M for RSVA/RSVB^b^
FAO	–	M	–	–
GX^c^	E; N2; RdRp	M; PA; PB2	M; NSP	N for RSVA/RSVB
TP^d^	N; S	M	M	N for RSVA, M for RSVB
PC^c^	RdRp	M	NP	N for RSVA/RSVB

SARS-CoV-2, severe acute respiratory syndrome coronavirus 2; IAV, Influenza A virus; IBV, Influenza B virus; RSVA/B, respiratory syncytial virus A/B; SoC, standard of care; FAO, Food and Agriculture Organization of United Nations; GX, Xpert^®^ Xpress SARS-CoV-2, Flu, RSV Kit; TP, TaqPath™ COVID-19, FluA/B, RSV Combo Kit; PC, PowerChek™ SARS-CoV-2, Influenza A&B, RSV Multiplex Real-time PCR Kit II; E, Envelop; M, Matrix; N, nucleocapsid; NP, nucleoprotein; NSP, non-structural protein; PA, polymerase acidic protein; PB2, polymerase basic protein; RdRp, RNA-dependent RNA polymerase; S, spike. MPAs target one, two or three genes for detection of each SARS-CoV-2, IAV, IBV and RSV.

a SoC use two sets of primers and probes performed in two separate wells/PCR runs for each sample for detection of SARS-CoV-2.

b SoC use different optical channels to detect the RSV targets and then provide separate results for RSVA and RSVB.

c GX and PC use separate optical channels to detect SARS-CoV-2, IAV, IBV and RSV and provide results for each virus separately.

d TP uses one optical detection channel for the detection of IAV and IBV and provides a combined result for influenza A/B, and similarly one optical channel is used for detection of RSVA and RSVB providing a combined result for RSV.

Standard of care assays (SoC) utilized at IPC for the detection of SARS-CoV-2 (CoV-SoC), influenza A virus (IAV-SoC), influenza B virus (IBV-SoC) and RSV (RSV-SoC), consisting of single RT-PCR tests (Table 1), served as reference (27–30). In addition, IAV samples were tested using Food and Agriculture Organization of United Nations (FAO) recommended primers and probes developed by the Australian Center for Disease Prevention for the detection of M gene from avian influenza viruses (AIV) in Asia (31). All SoC and FAO were performed on a CFX96™ instrument and results analyzed with the corresponding software.

### 2.2. Study specimens

In-house Cambodian viral isolates, including several variants of SARS-CoV-2 and subtypes of human seasonal influenza, and AIV (Table 2) were heat-inactivated and used to assess the limit of detection (LoD) of each assay. For each isolate, a serial-dilution was prepared in standard Viral Transport Media (VTM) and stored at −70°C. Immediately after thawing, 300 μl of sample was tested with GX and 400 μl was extracted with the MagMAX™ Viral/Pathogen II Nucleic Acid Isolation Kit on a KingFisher Flex system (Thermo Fisher Scientific), using the volume recommended by TP instructions for use, and RNA eluted with 50 μl nuclease-free water. Each 10-fold dilution was tested in triplicate with SoC. End-point dilution was defined as lowest dilution at which all replicates were positive. Subsequently, each viral isolate was tested with GX, TP, PC and SoC in parallel on the same day, at the previously determined end-point dilution and a minimum of two half-log_10_ dilutions on either side of the LoD.

**Table 2:** Comparison of limit of detection between evaluated and standard assays.

Virus	Host	Subtype	Lineage	FAO	GX	TP	PC
SARS-CoV-2	Human	Wuhan	Indian, B.6, 2000	Not done	0	0	0
		Alpha	2021	Not done	−1	**1**	**1**
		Omicron	BA.2, 2022	Not done	0	0	0
Influenza	Human seasonal influenza	A/H1N1	pdm, 2019	−2	−1	**1**	**1**
		A/H3N2	2019	**1**	0	0	**1**
		A/H3N2	2022	0	−1	0	0
		B/Vic	Victoria	Not done	**1**	0	**1**
		B/Yam	Yamagata	Not done	**1**	**2**	**1**
	Avian influenza in human cases	A/H5N1	2.3.2.1c 2014	0	**2**	**1**	**1**
		A/H9N2	G9/BJ94 2021	**1**	0	**1**	**2**
	Avian influenza in poultry samples	A/H5N1	2.3.2.1c 2021	0	0	0	**1**
		A/H5N8	2.3.4.4b 2022	0	0	0	0
		A/H7N4	Jiangsu 2018	0	−1	−1	0

FAO, Food and Agriculture Organization of United Nations recommended primers and probes developed by the Australian Center for Disease Prevention for the detection of M gene from avian influenza; GX, Xpert^®^ Xpress SARS-CoV-2, Flu, RSV Kit; TP, TaqPath™ COVID-19, FluA/B, RSV Combo Kit; PC PowerChek™ SARS-CoV-2, Influenza A&B, RSV Multiplex Real-time PCR Kit II; SARS-CoV-2, severe acute respiratory syndrome coronavirus 2. The table presents the difference between SoC and evaluated assay in term of Log10 dilution. Delta LoD resulted in 0 if MPA and SoC had the same LoD (in light green), ≥1 if LoD of MPA was lower than SoC (in dark green, bold) and <0 if LoD of MPA was higher than SoC (in red). Delta LoD resulted in 0 if MPA and SoC had the same LoD, and <0 if LoD of MPA was higher than SoC.

To assess clinical accuracy, residual URT specimens collected in VTM were selected based on routine results obtained under public health surveillance for influenza (IAV *n* = 84, IBV *n* = 5) and RSV (*n* = 32), and pandemic response for SARS-CoV-2 (*n* = 58), upon availability and volume of stored samples (Supplementary Table S1). Different lineages were selected based on molecular and sequencing results. Samples previously tested negative for all targets were also included (*n* = 126). Similar to viral isolates, 300 μl of sample were used for GX testing and 400 μl for extraction. Extracted RNA served for side-by-side testing with TP, PC, and SoC, performed on the same day. As amount of RNA for each sample was limited to re-test with SoC, routine negative results were utilized for comparison in the following cases: for IAV, IBV, and RSV among the SARS-CoV-2 samples; for IBV and RSV for IAV samples; for IAV and RSV for IBV samples; for SARS-CoV-2 and IAV/IBV among negative samples. Influenza and RSV samples collected during influenza/RSV seasons in 2016–2019 were negative for SARS-CoV-2. However, if one targeted virus was detected with any MPA, the related SoC was performed using the same RNA. For 31/84 IAV specimens, remaining volume was not sufficient to perform GX testing in addition to extraction.

### 2.3. Statistical analysis

For each assay, individual cycle threshold (Ct) values (Ct-values) and interpretation as positive or negative according to test cut-off were recorded for each viral isolate and clinical sample. Three (SARS-CoV-2; influenza; RSV) results for TP or 4 (SARS-CoV-2; IAV; IBV; RSV) for GX, PC and SoC were provided for each sample. Comparison was performed for each virus individually. Difference between LoD with SoC and each MPA (D-LoD) was calculated for each viral isolate. The difference in LoD (D-LoD) was expressed as log_10_ dilution of LoD of SoC minus log_10_ dilution of MPA. D-LoD resulted in 0 when MPA and SoC had the same LoD, in D-LoD ≥ 1 if MPA LoD was lower than SoC (MPA performed better than SoC) and in D-LoD < 0 if MPA LoD was higher than SoC (MPA performed worse than SoC). Sensitivity, specificity, positive and negative predictive values (PPA/NPA) were calculated using STATA statistical software (v12.1, College Station, TX, United States). Overall accuracy to detect viruses in clinical samples for GX, TP and PC was assessed by percent agreement, corresponding to the proportion of identical results between each MPA evaluated and SoC for each virus, and 95% confidence intervals (95% CI).

### 2.4. Assessment of utility

Total turnaround time per specimen, including extraction, RT-PCR, and interpretation of results were compared. Cost comparison accounted for reagents and shipments to Cambodia at current pricing structures. Other criteria to help drive choice for suitability included the volume of sample for extraction/assay, amount of RNA for RT-PCR, equipment requirements, practicability of interpretation software, result type obtained for each targeted virus.

### 2.5. Ethical approval statement

This study was approved by the Cambodian National Ethics Committee for Health Research (N°050 NECHR, 2022). Since samples were obtained as part of the national influenza surveillance system and as part of outbreak response for SARS-CoV-2, requirement for informed consent was waived for their use in the study. All samples were de-identified and the database contained no patient information.

## 3. Results

### 3.1. Limit of detection

The three MPAs consistently detected all selected viral strains with a difference in LoDs (D-LoDs) ranging from −2 to +2 Log10 dilutions according to strains and assays (Table 2). A higher D-LoD occurred on GX for 4/13 isolates: SARS-CoV-2 Alpha variant and recent A(H1N1), A(H3N2–2022) IAV and A(H7N4) AIV from poultry sample, indicating a lower sensitivity of the GX MPA compared to the SoC assay. However the LoD was equivalent or lower for other virus isolates. The TP MPA had equivalent (for 7/13 isolates) or lower LoD (for 5/13 isolates) compared to SoC assay except for A(H7N4). For the PC assay, all LoDs were equivalent or marginally lower than SoC. Overall, the MPAs showed similar LoDs than the respective SoC assays indicating a comparable sensitivity.

### 3.2. Performance on clinical samples

**Figure 1:**
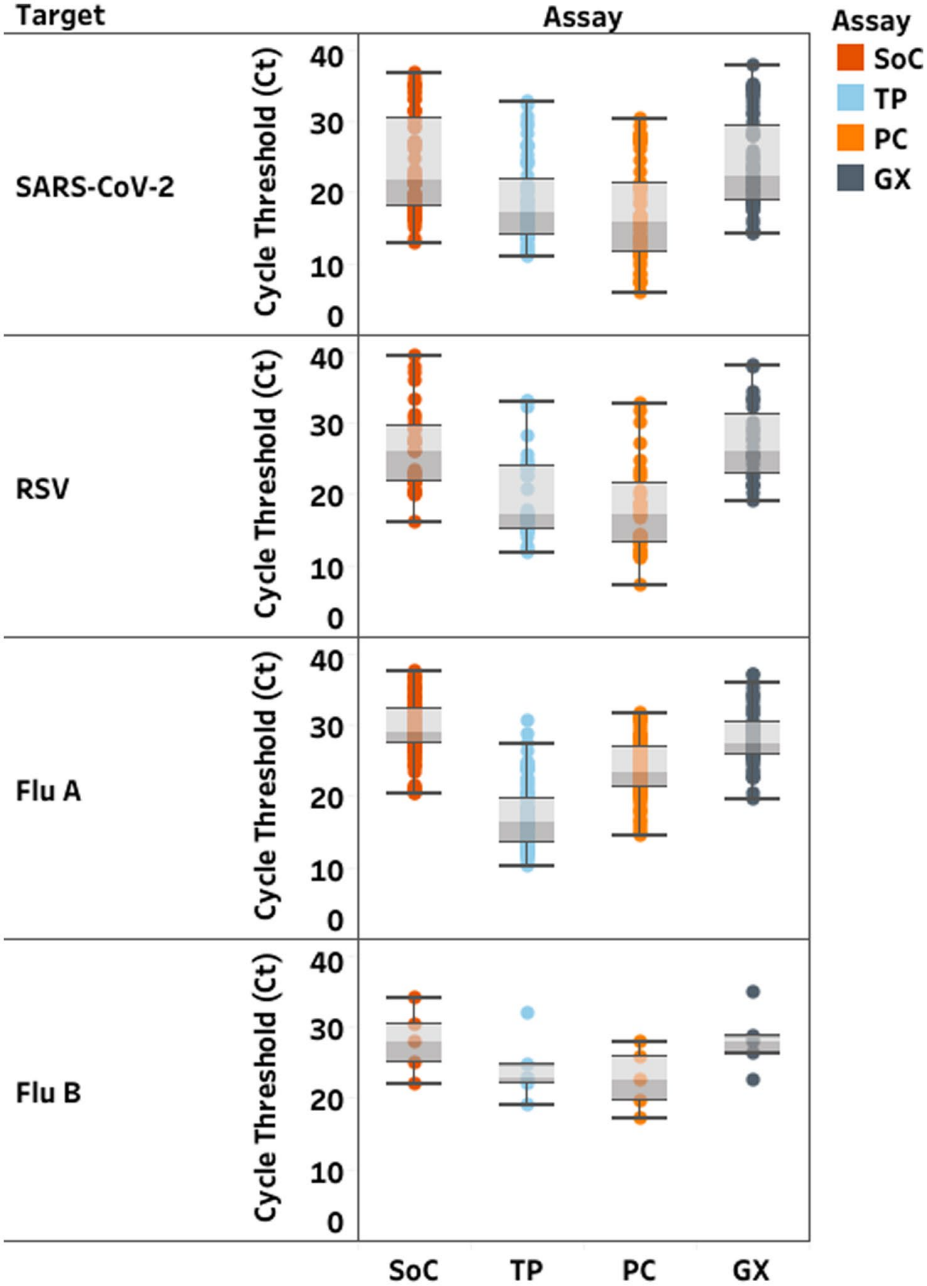
Distribution of cycle threshold (Ct)-values (median; min-max) in clinical samples according to each RT-PCR assay. Standards of Care (SoC) are displayed in dark orange, Thermofisher TaqPath™ COVID-19, FluA/B, RSV Combo Kit (TP) in light blue, Kogene PowerChek™ SARS-CoV-2, Influenza A&B, RSV Multiplex Real-time PCR Kit II (PC) in light orange, Cephied Xpert^®^ Xpress SARS-CoV-2, Flu, RSV Kit (GX) in grey. Ct values are displayed for severe acute respiratory syndrome coronavirus 2 (SARS-CoV-2), respiratory.

Median and range of Ct-values on GX were equivalent to SoC, but lower using TP and PC (Figure 1). TP and PC adequately detected all selected positive samples from all lineages and all negative samples (Supplementary Table S2) with sensitivity and specificity over 95%. GX identified all but four samples, for which the test failed to detect RSV. Discordant results occurred in 14 samples on the remaining targets (Supplementary Table S3). Among two samples with RSV/SARS-CoV-2 co-infection, none of the MPAs detected SARS-CoV-2 in the first, and PC failed to detect SARS-CoV-2 in the second sample. Among the 4/32 RSV samples (12.5%) not detected with GX, three were mono-infections. The last one had an IAV/RSV co-infection. TP also failed to detect RSV in this sample. Eight additional samples had a positive result for one target but were not detected by SoC or other MPA: two had positive result only with TP (one for influenza; one for RSV) and six only with PC (one for SARS-CoV-2; one for IAV; three for IBV; one for RSV).

Overall, positive and negative predictive values (PPV, NPV) ranged between 97 and 100%, except for detection of IBV using PC, which dropped to 62.5%. However, overall accuracy between SoC and MPA ranged between 97 and 100% of agreement (Table 3).

**Table 3:** Comparison of evaluated assay and standard WHO/GIRS assays currently used in the laboratory.

		Sensitivity	Specificity	Positive predictive value	Negative predictive value	Overall percent agreement
SARS-CoV-2	GX	96.6 (88.3–99.6)	100.0 (93.7–100.0)	100.0 (97.8–100.0)	98.8 (95.7–99.9)	99.1 (97.0–99.9)
	TP	96.6 (88.3–99.6)	100.0 (98.0–100.0)	100.0 (93.7–100.0)	98.9 (96.0–99.9)	99.2 (97.0–99.9)
	PC	95.0 (85.9–98.9)	99.4 (96.9–100.0)	98.2 (90.6–100.0)	98.4 (95.3–99.7)	98.3 (95.8–99.5)
Influenza A virus	GX	100.0 (93.3–100.0)	100.0 (98.0–100.0)	100 (93.3–100.0)	100.0 (98.0–100.0)	100.0 (98.4–100.0)
	PC	100.0 (95.7–100.0)	99.4 (96.9–100.0)	98.8 (93.6–100.0)	100.0 (98.1–100.0)	99.6 (98.0–100.0)
Influenza B virus	GX	100.0 (47.8–100.0)	100 (98.2–100.0)	100.0 (47.8–100.0)	100.0 (98.2–100.0)	100.0 (98.2–100.0)
	PC	100.0 (47.8–100.0)	98.6 (96.1–99.7)	62.5 (24.5–91.5)	100.0 (98.3–100.0)	98.7 (96.2–99.7)
InfluenzaA/B	TP^a^	100.0 (96.0–100.0)	100.0 (98.0–100.0)	100.0 (97.9–100.0)	100.0 (97.9–100.0)	100.0 (98.6–100.0)
Respiratory syncytial virus	GX	87.5 (71.0–96.5)	100.0 (97.8–100.0)	100.0 (87.7–100.0)	97.6 (94.0–99.3)	97.9 (94.8–99.4)
	TP	96.9 (83.8–99.9)	100.0 (97.9–100.0)	100.0 (88.8–100.0)	99.4 (96.9–100.0)	99.5 (97.4–100.0)
	PC	100.0 (89.1–100.0)	99.4 (96.9–100.0)	97.0 (84.2–99.9)	100.0 (97.9–100.0)	99.5 (97.4–100.0)

SARS-CoV-2, severe acute respiratory syndrome coronavirus 2; GX, Xpert^®^ Xpress SARS-CoV-2, Flu, RSV Kit; TP, TaqPath™ COVID-19, FluA/B, RSV Combo Kit; PC, PowerChek™ SARS-CoV-2, Influenza A&B, RSV Multiplex Real-time PCR Kit II; FAO, Food and Agriculture Organization of United Nations recommended primers and probes developed by the Australian Center for Disease Prevention for the detection of M gene from avian influenza.

a TP provides a combined influenza result for IAV and IBV as targets are combined in the same optical detection channel. Therefore, IAV and IBV results were combined for statistical tests.

### 3.3. Assessment of utility

MPAs provide results for detection of SARS-CoV-2, influenza, and RSV in a single RT-PCR assay compared to five SoC RT-PCR reactions to get the same information, with variable costs, testing burden, and implementation parameters (Table 4). The decision of what MPA to choose depends on the laboratory setting (available equipment and personnel) and circumstances like available sample volume and demand for fast testing turnover. Manufacturer instructions for GX and TP have strictly defined volume of sample/elution and RNA, whereas PC, similar to SoC, allows the use of different sample volumes according to extraction kit. A hindrance might be that GX and TP are designed for manufacturer-specific instruments, as SoC and PC can be utilized on any instrument providing more than two and four optical channels, respectively. Run time of 90 min per run for 94 samples with TP and PC is similar to SoC but simultaneously provide results for all targets. In contrast, GX integrates the process from extraction to result, but only for one sample per run. TP required specific training to use the QuantStudio 5 and CE-IVD software for interpretation, while SoC and PC were interpreted on current laboratory software.

**Table 4:** Comparison of multiplex assays with regards to test specifications, costs and accomplishment.

	Standard assay (SoC)	TaqPath (TP)	Powerchek (PC)	Xpert Xpress (GX)
Manufacturer	–	Thermo Fisher Scientific	Kogene Biotech	Cepheid
Pathogen detection	SARS-CoV-2, IAV, IBV, RSVA and RSVB	SARS-CoV-2, InfluenzaA/B, RSV	SARS-CoV-2, IAV, IBV, RSV	SARS-CoV-2, IAV, IBV, RSV
Number of PCR reactions	4	1	1	1
Sample volume	Not specified^a^	400 μl	Not specified^a^	300 μl
Elution volume	Not specified^a^	50 μl	Not specified^a^	Not applicable
RNA volume	25 μl (5 μl/each)	17.5 μl	5 μl	Not applicable
Internal control	Not provided	Provided	Provided	Provided in cartridge
Step to add IC	Extraction	Extraction	PCR mix	Not applicable
Number of samples tested on the same assay	93	94	94	1
Run on time	90 min	90 min	90 min	36 min
Time to result^a^	540 min^b^	145 min^b^	145 min^b^	40 min
Personnel training	Low	High	Low	Low
RT-PCR Instrument	Any with >2 optical channels	Applied Biosystems™ 7,500 Fast; QuantStudio™5; QuantStudio™ 7 Flex, 384–well block	Any with 4 optical channels	GenXpert Instrument
Interpretation of results with software	RT-PCR Instrument	Pathogen Interpretive	RT-PCR Instrument	GenXpert Instrument
Cost reagents per test (US$)	20^c^^,^^d^	25^e^	13.6^e^	31^f^

a Sample volume according to the extraction kit manufacturer manual for use.

b Extraction 35 min, PCR 90 min each, interpretation of results 10 min for SoC, 20 min for MPA.

c Providing that we perform 5 RT-PCR assays (2 PCR for SARS-CoV-2 (E and RdRP genes); 1 for IAV; 1 for IBV; and 1 for RSV) for Standard of care with 5 μl for each RT-PCR.

d We calculated a cost of 4 $ per test in Standard PCR assay.

e Shipment and controls in each PCR plate included.

f Shipment included. Prices provided by Singapore for TP, Korea for PC and France for GX.

## 4. Discussion

Incorporation of MPAs into routine surveillance of SARS-CoV-2, influenza and RSV is critical to expand pathogen detection while minimizing costs and constrain on human resources within existing capacities/capabilities. A side-by-side comparison of GX, TP and PC using the same large set of viral isolates, including avian influenza, and clinical samples was critical for evaluation, especially for limited resource settings with high probability of AIV spillover.

GX, TP and PC consistently detected all viral lineages of SARS-CoV-2 and influenza; however, GX had slightly higher LoD compared to SoC. Decreased GX testing volume compared to extraction possibly contributed to this discrepancy. Each MPA demonstrated high accuracy to detect all viruses in clinical samples. Overall, median and range of Ct-values obtained with TP and PC were lower than with SoC and GX. Differences in sample volume and lower number of samples tested with GX could affect these values. Discrepancies between assays did occur. One SARS-CoV-2 infection was not detected by PC, and one and four RSV were not detected by TP and GX, respectively. Low viral load (Ct = 38–39) by SoC close to LoD and storage issues could impair detectability. Difference in sample testing volume could impact detection with GX. Unfortunately, remaining sample volume did not allow repeated GX testing. Eight samples had a positive result for one target, but were negative with SoC and other MPAs and were considered as false positive results.

Most commercial tests are not specifically designed to identify/distinguish AIV or novel IAV. However, detection of zoonotic AIV infection is paramount, especially in endemic countries such as Cambodia (32), and for pandemic prevention and preparedness globally. GX package insert does assert the test adequately detects AIV (24); however, PC and TP have no previous data available. This study indicates MPAs can likely identify AIV cases with high accuracy to detect all targets in clinical samples. All variants of SARS-CoV-2 circulating in Cambodia during the collection period were detected.

Previous evaluations of GX reported a high concordance using retrospective clinical samples compared to other Cepheid assays and several MPAs. In the United Kingdom (17), Netherlands (33), and Hong Kong (34), GX had 95–99.64% PPA and 100% NPA for targets compared to SoC. No false positive results were observed with GX in this study, but some were previously reported for SARS-CoV-2/RSV co-infections (33, 34). A previous version of PC was evaluated in South Korea with 100% PPA/NPA for SARS-CoV-2, IAV, and IBV and 93.1%/100% for RSV versus comparator (35). TP has been evaluated using nasopharyngeal specimens with PPA/NPA at 98.2%/100, 100%/96.5, and 98.2%/92.8% for SARS-CoV-2, influenza, and RSV, respectively, compared to reference assays (25). Detection accuracy in the present study of 97–100% PPA for all targets is similar to these previous findings.

In addition to detection efficiency, MPAs’ utility is critical for routine use in laboratories. Each GX cartridge only tests one sample at-a-time and is more expensive than other MPAs. However, GX provides fastest results with minimal sample handling, an advantage for emergency cases, reduced sample loads, and/or restricted human resources. Moreover, GX does not require extensive expertise in techniques or interpretation. TP and PC minimize volume of RNA required, and significantly reduce instrument occupation time, potentially critical during periods with high testing demand. Result interpretation is provided automatically using specific software for TP and GX, with TP requiring review of amplification curves (25). PC and SoCs require user interpretation, allowing flexibility but also need expertise to avoid misinterpretation and introduction of potential technical error. Based on the similarities and differences of the SoC and MPAs (Table 4), choice of MPA should be made based on current circumstances, restrictions, and demand.

A prospective design was not possible in this study and retrospective investigation was conducted on stored samples, potentially resulting in selection bias and reduced sample quality. This impact was probably limited by selection based on available volume versus specific viral characteristics. Sample volume and/or extracted RNA was too limited to repeat all SoC for all samples, thus some routine results were included from time of reception. However, if any targeted virus was detected with MPA, the same extracted RNA was retested with corresponding SoC. A few samples with low viral loads and limited IBV sample number restricted some further investigations. Finally, determination of LoD by viral copy number requires extensive *in vitro* assessment and electron microscopy, which is not readily available in Cambodia. Future experiments with tittered viral isolates will add to the assessment of LoD.

The reality of overlapping clinical presentations of concurrently circulating viruses, funding and reagent constraints, and limited human resources require integration of MPAs into routine VRI surveillance. Timely diagnosis decreases unnecessary laboratory testing, minimizes use of antibiotics, and maximizes effectiveness of measures to control infection. Appropriate and early antiviral treatment reduces complications, hospitalizations, and mortality (36). Simultaneous detection of SARS-CoV-2, influenza, and RSV in a single test accelerates time from sampling to diagnosis, and can utilize capacity/capability developed during the COVID-19 pandemic. MPAs also preserve consumables, and streamline human resources to respond to other endemic or emerging pathogens. As result, MPAs have the potential to sustain and even expand surveillance systems, thereby strengthening understanding of seasonal pathogens, availability for vaccine development, and epidemic/pandemic preparedness, prevention, and response.

## Data availability statement

The raw data supporting the conclusions of this article will be made available by the authors, without undue reservation.

## Ethics statement

The studies involving human participants were reviewed and approved by Cambodian National Ethics Committee for Health Research. Written informed consent for participation was not required for this study in accordance with the national legislation and the institutional requirements.

## Author contributions

JF, MG, OO, CU, and HA: resources. NB, KC, LH, SK, KS, SI, PC, BC, LK, and CF: investigation. NB and CF: data curation. NB, JF, MG, OO, CU, and EK: conceptualization. KC, VH, and EK: supervision. NB, CF, and EK: writing – original draft. NB, HA, EK, JF, MG, OO, and CF: writing – review and editing. All authors contributed to the article and approved the submitted version.

## Funding

This work was supported by Thermo Fisher Scientific, who loaned the QuantStudio 5™ RT-PCR, 96 well, 0.2 ml instrument (Applied Biosystems) and laptop to IPC for the purpose of the study, provided TaqPath™ COVID-19, FluA/B, RSV Combo Kits, and co-authors included were involved in the study design, analysis and interpretation of TaqPath results and reviewed the report. French Agency for Development funded the ECOMORE II project and a COVID-19 top-up (project no. CZZ 2146 01A), providing payed salary to NB for a senior medical virologist position under a temporary contract with the Institut Pasteur du Cambodge. HA is supported by the German Centre for International Migration and Development (CIM). Influenza, COVID-19, and RSV work at IPC and EK are supported, in part, by the World Health Organization and the Food and Agriculture Association of the United Nations.

## Conflict of interest

JF, MG, OO, and CU were employed by Thermo Fisher Scientific.

The remaining authors declare that the research was conducted in the absence of any commercial or financial relationships that could be construed as a potential conflict of interest.

## Publisher’s note

All claims expressed in this article are solely those of the authors and do not necessarily represent those of their affiliated organizations, or those of the publisher, the editors and the reviewers. Any product that may be evaluated in this article, or claim that may be made by its manufacturer, is not guaranteed or endorsed by the publisher.
